# The Association between Serum Serine and Glycine and Related-Metabolites with Pancreatic Cancer in a Prospective Cohort Study

**DOI:** 10.3390/cancers14092199

**Published:** 2022-04-28

**Authors:** Hung N. Luu, Pedram Paragomi, Renwei Wang, Joyce Y. Huang, Jennifer Adams-Haduch, Øivind Midttun, Arve Ulvik, Tin C. Nguyen, Randall E. Brand, Yutang Gao, Per Magne Ueland, Jian-Min Yuan

**Affiliations:** 1 UPMC Hillman Cancer Center, University of Pittsburgh Medical Center, 5150 Centre Avenue, Suite 4C, Pittsburgh, PA 15232, USA; paragomi@pitt.edu (P.P.); wangr2@upmc.edu (R.W.); yonhuang@utmb.edu (J.Y.H.); adamshaduchj@upmc.edu (J.A.-H.); brandre@upmc.edu (R.E.B.); yuanj@upmc.edu (J.-M.Y.); 2 Department of Epidemiology, School of Public Health, University of Pittsburgh, 130 De Soto St, Pittsburgh, PA 15261, USA; 3 Bevital A/S, Jonas Lies Veg 87, 5021 Bergen, Norway; bjorn.midttun@med.uib.no (Ø.M.); per.ueland@ikb.uib.no (P.M.U.); 4 Department of Clinical Science, University of Bergen, Postboks 7804, 5020 Bergen, Norway; arve.ulvik@uib.no; 5 Department of Computer Science and Engineering, University of Nevada at Reno, Reno, NV 89557, USA; tinn@unr.edu; 6 Division of Gastroenterology, Hepatology and Nutrition, Department of Medicine, University of Pittsburgh, Pittsburgh, PA 15213, USA; 7 Department of Epidemiology, Shanghai Cancer Institute, Renji Hospital, Shanghai Jiaotong University School of Medicine, Shanghai 201112, China; ytgao@vip.sina.com; 8 Laboratory of Clinical Biochemistry, Haukeland University Hospital, 5021 Bergen, Norway

**Keywords:** pancreatic cancer, risk factors, serine, glycine

## Abstract

**Simple Summary:**

Serine and glycine have an important role in the folate-dependent one-carbon metabolism. No prior epidemiologic study has evaluated the associations for serum levels of serine and glycine with pancreatic cancer risk. We performed a nested case-control study of 129 incident pancreatic cancer cases and 258 individually matched controls within the Shanghai Cohort Study, a prospective cohort study involving 18,244 male residents in Shanghai, China. We found that the risk of pancreatic cancer was reduced by more than 70% in individuals with elevated levels of serine and glycine in serum collected, on average, more than 10 years prior to cancer diagnosis. These novel findings support a protective role of serine and glycine against the development of pancreatic cancer in humans that might have an implication for pancreatic cancer prevention.

**Abstract:**

Background. Serine and glycine play an important role in the folate-dependent one-carbon metabolism. The metabolism of serine and glycine has been shown to be associated with cancer cell proliferation. No prior epidemiologic study has investigated the associations for serum levels of serine and glycine with pancreatic cancer risk. Methods. We conducted a nested case-control study involved 129 incident pancreatic cancer cases and 258 individually matched controls within a prospective cohort study of 18,244 male residents in Shanghai, China. Glycine and serine and related metabolites in pre-diagnostic serum were quantified using gas chromatography-tandem mass spectrometry. A conditional logistic regression method was used to evaluate the associations for serine, glycine, and related metabolites with pancreatic cancer risk with adjustment for potential confounders. Results: Odds ratios (95% confidence intervals) of pancreatic cancer for the highest quartile of serine and glycine were 0.33 (0.14–0.75) and 0.25 (0.11–0.58), respectively, compared with their respective lowest quartiles (both *p*’s < 0.01). No significant association with risk of pancreatic cancer was observed for other serine- or glycine related metabolites including cystathionine, cysteine, and sarcosine. Conclusion. The risk of pancreatic cancer was reduced by more than 70% in individuals with elevated levels of glycine and serine in serum collected, on average, more than 10 years prior to cancer diagnosis in a prospectively designed case-control study. These novel findings support a protective role of serine and glycine against the development of pancreatic cancer in humans that might have an implication for cancer prevention.

## 1. Introduction

Pancreatic cancer is ranked as the 12th most common cancer in men and 11th most common cancer in women, with an estimated 460,000 new cases in 2018 worldwide [1]. In the United States, pancreatic cancer is the third leading cause of cancer death with an estimated 47,050 deaths due to pancreatic cancer in 2020 [2]. The prognosis of pancreatic cancer is poor; the five-year survival rate is only 8% of patients after diagnosis [3]. In addition, the incidence and mortality rates of pancreatic cancer have been increasing during past four decades [4]. In China, between 1990 and 2019, there were 1,817,952 incident cases and 1,854,761 deaths of pancreatic cancer [5]. Overall, the incidence rates of pancreatic cancer in China increased annually among those aged 25 years old and older. The annual percentage change of pancreatic cancer incidence between 1990 and 2019 in China was 2.3 (95% confidence interval-CI: 2.1–2.5) [6]. Established risk factors for pancreatic cancer are cigarette smoking, alcohol consumption, obesity, chronic pancreatitis, and type 2 diabetes [4,7]. Collectively, less than half of the pancreatic cancer burden is attributable to these identified risk factors [8]. The underlying causes are still controversial and unknown for majority of pancreatic cancer cases. Furthermore, the mechanistic networks implicated in pancreatic carcinogenesis are not fully understood. Therefore, it is an urgent need to identify novel etiological factors, especially modifiable factors that would help develop an evidence-based strategy for primary prevention of pancreatic cancer.

**Figure 1:**
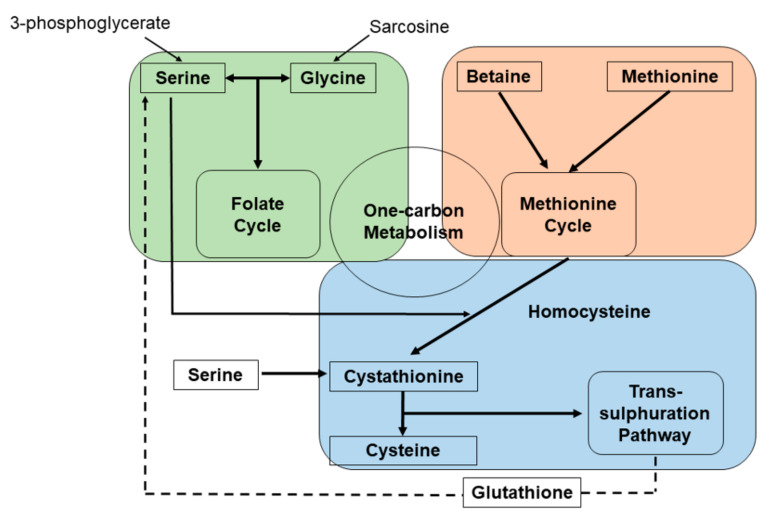
Schematic diagram of serine and glycine metabolism pathways.

One-carbon metabolism involves the folate cycle and the methionine cycle. These cycles are responsible for the integration of different nutritional sources (i.e., amino acids, glucose and vitamins) and for the generation of diverse physiologically functional compounds (i.e., nucleotides and proteins). These one-carbon metabolic cycles also provide the substrates for methylation reactions and maintenance of redox status [9]. The two one-carbon cycles are interconnected. 3-Phosphoglycerate (3PG), which is an intermediate metabolite involved in glycolysis, can be converted into serine, a major donor of carbon to the folate cycle [10,11]. The carbon donation of serine is linked to the conversion of tetrahydrofolate (THF) to methylene-tetrahydrofolate (Methylene-THF) [12,13]. Glycolysis, a determinant for this pathway, is known to be associated with cancer initiation and progression in rat carcinoma models [12,14,15]. Another modular unit of one-carbon metabolism is the trans-sulfuration pathway, which is connected to the methionine cycle through the intermediate homocysteine [9]. Accordingly, cystathionine is generated from serine, which can condense enzymatically with homocysteine catalyzed by cystathionine beta-synthase (CBS) and plays an essential role for redox buffering (Figure 1). Both homocysteine and serine are precursors for cysteine synthesis (Review in Yang et al. [16]).

Evidence from prior experimental studies has shown that the serine-glycine metabolism has been associated with cancer cell proliferation and survival [17,18,19]. For example, Jain et al. [19] found the mitochondrial glycine biosynthetic pathway is strongly associated with proliferation rates of multiple cancer cells whereas serine deprivation was associated with slowed cancer cell proliferation, which may be due to the depletion of purine synthesis and decreased adenosine triphosphate (ATP) production [20,21]^,^. Serine starvation triggers de novo synthesis of this amino acid which by utilizing glycolytic intermediates, impedes energy production [22].

Prior studies have found elevated levels of serum serine and glycine in cases of various sites of cancers including the lung [23], bladder [24], prostate [25], kidney [26], and colon [27] whereas other studies produced null results in rectal cancer [27] and pancreatic cancer [28]. For example, Jiao et al. [28] found no significant difference in serum concentrations of serine and glycine between pancreatic cancer cases and controls within the Women’s Health Initiative Study, probably due to a modest sample size (only 30 pancreatic cancer cases vs. 30 matched controls). On the contrary, in a study of 136 female non-smoking subjects (i.e., 65 patients with non-small cell lung carcinoma, 6 with benign lung tumor and 65 healthy controls), Mu et al. [23] found that the serum concentrations of both serine and glycine in lung cancer patients were significantly lower than in healthy controls. Similarly, a large case-control study involving 3000 prostate cancer patients and 3000 controls within the JANUS Cohort in Norway reported a significantly lower risk of prostate cancer (odds ratio-OR = 0.83, 95% confident interval-CI: 0.70–1.00) associated with higher level of glycine [25]. Furthermore, in a study of 58 cases of renal cell carcinoma versus 59 controls, Mustafa et al. [26] reported that serum levels of both serine and glycine in renal cell carcinoma patients were significantly lower than those in controls. In another study of 59 colon cancer patients and 58 controls, Leichtle et al. [29] found that serum glycine level was significantly higher in controls than colon cancer patients while there was no significant different between colon cancer cases vs. control in serum serine level.

One-carbon metabolism cycles are complex metabolic network with several chemical catalysts involved. Better understanding of this milieu provides ample opportunities for risk prediction and potential prevention. The objective of the current analysis was, therefore, to comprehensively examine these associations in a prospective cohort study of more than 18,000 study participants.

## 2. Methods

### 2.1. Study Population

The present study was based on the Shanghai Cohort Study that has been previously described [30]. The Shanghai Cohort Study is an on-going prospective cohort study that enrolled 18,244 male residents at 45–64 years of age who resided within four communities in the city of Shanghai, China, from January 1986 to September 1989. At the time of enrolment, a trained nurse interviewed each study participant in-person. Information on use of tobacco and alcohol consumption, usual dietary pattern and past medical history were collected via an interview. At the end of the interview, the nurse also collected a 10-mL non-fasting blood sample and a single-void urine sample from the study participant. Serum and urine samples were stored at −72 °C. The study protocol has been approved by the Institutional Review Boards (IRBs) of the University of Pittsburgh, and Shanghai Cancer Institute. The present study was also approved by the IRB of the University of Pittsburgh.

### 2.2. Case Ascertainment of Pancreatic Cancer

The incident cases of cancer and death among participants of the Shanghai Cohort Study were identified through annual follow-up interviews on all surviving study participants or next of kin for those deceased in the preceding 12 months. In addition, an annual record linkage analyses were conducted with the databases of the population-based Shanghai Cancer Registry and the Shanghai Municipal Vital Statistics Office to identify and confirm new cases of cancer and deaths, respectively. Pancreatic cancer was defined per the International Classification of Diseases-Oncology, 9th edition (ICD-9) code 157. In the most recent follow-up data in 2015, 3.7% of original cohort participants were lost to the follow-up and 3.3% declined to participate in the continued follow-up interview. Since their cancer incidence and/or vital status had been updated using the linkage analyses described above, the follow-up for occurrence of cancer and death among all cohort participants was virtually complete.

### 2.3. Nested Case-Control Study

The current analysis was a nested case-control study within the Shanghai Cohort Study in which 129 incident pancreatic cancer cases were detected as of 31 December 2009. For each pancreatic cancer case, two control subjects were randomly selected among the eligible participants of the Shanghai Cohort Study who were free of cancer and alive during the time period from the phlebotomy to the timepoint of diagnosis of pancreatic cancer of the index case. The selected controls were individually matched to the index case by age (±2 years), date of blood draw (±one month), and the neighborhood of residence at the time of enrolment.

### 2.4. Assessment of Serum Biomarkers

Serum specimens of cases and their matched controls were processed, aliquoted, shipped in frozen state and assayed together at Bevital A/S, Bergen, Norway (www.bevital.no, Accessed date: 15 December 2015). The serum samples of each matched case-control set (1 case and 2 controls) were placed next to each other in a random order and tested simultaneously. The personnel were blinded to the case/control status of the test samples.

Gas chromatography-tandem mass spectrometry (LC-MS/MS) [31] was used to quantify serum serine, glycine, sarcosine, glutamine, total cysteine, cystathionine and methionine, while liquid chromatography-tandem mass spectrometry was applied to measure creatinine, choline, betaine [32], cotinine [33], and pyridoxal 5′-phosphate (PLP) [33]. Serum creatinine was used for the calculation of glomerular filtration rate (eGFR), an indicator of renal function [34]. Serum cotinine is a metabolite of nicotine, and is utilized as an indicator of recent exposure to tobacco smoking and/or use of other nicotine-containing products [35]. For quality control purposes, 14 duplicated samples derived from a pool of serum samples collected from cohort participants at the same period of the study sample collection were dispersed in seven batches of test sample (two per batch). The within-batch coefficients of variation (CV) for all biomarkers tested ranged between 0.7% and 5.0% while the between-batch CV ranged between 1.3% and 2.6% (Table S1).

### 2.5. Statistical Analysis

We used natural logarithmic transformation of original values in order to reduce their skewness and also improve the normality of their distributions. To examine the difference in concentrations between cases and controls according to demographic characteristics we used the analysis of covariance (ANCOVA).

The conditional logistic regression method was used to calculate odds ratios (ORs) and the 95% confidence intervals (CIs) for pancreatic cancer according to quartiles of serum biomarkers studied, the cut-offs of which were determined by their distributions among control subjects. To adjust for the potential confounding effects, multivariable logistic models included level of education (no formal schooling, primary school, secondary school or above), body mass index (BMI) (<18.5, 18.5–<23, or ≥23 kg/m^2^), smoking status (never, former, or current smokers), serum cotinine (nmol/L), alcohol consumption (based on number of drinks per week), history of diabetes (yes, no), serum PLP levels (nmol/L) [36], eGFR [36] and total methyl donor (i.e., sum of choline, betaine and methionine) [37]. The linear trend test was based on the ordinal values of quartile (i.e., 1, 2, 3, and 4) for each of the studied biomarkers with the risk of developing pancreatic cancer. We conducted sensitivity analysis stratified by median follow-up time (<13 versus ≥13 years).

Statistical analyses were performed using SAS software version 9.4 (SAS Institute, Cary, NC, USA). All *p* values reported are two-sided. *p* less than 0.05 was considered as statistically significant.

## 3. Results

The mean (standard deviation (SD)) age at blood draw for pancreatic cancer cases and controls were 56.4 (5.5) years and 56.5 (5.5) years, respectively. Among cases, the average (range) time interval from blood draw to cancer diagnosis was 12.5 years (3 months-23.2 years).

The baseline characteristics and selected risk factors of study participants are presented in Table 1. Compared with controls, pancreatic cancer cases had significantly higher percentage of current smokers and higher levels of serum cotinine but lower levels of total methyl donors. There were no statistically significant differences between cases and controls in age, BMI, eGFR, PLP, education level, alcohol intake and history of diabetes.

The serum concentrations of serine and glycine were significantly lower in pancreatic cancer cases than in controls (both *p’s* < 0.02; Table 2). There was no statistically significant difference in serum concentrations of sarcosine, cysteine, and cystathionine between cases and controls.

Higher levels of serine and glycine were significantly associated with decreased risk of pancreatic cancer (Table 3). Compared with the lowest quartile, the ORs (95% CIs) of pancreatic cancer for the highest quartile of serine and glycine were 0.33 (0.14–0.75) and 0.25 (0.11–0.58), respectively (both *p*_trend_ < 0.005). No statistically significant association was observed for risk of pancreatic cancer with any other biomarkers tested including cystathionine, cysteine, or sarcosine (Table 3).

We also examined the joint effect of serine and glycine on risk of pancreatic cancer. Compared with lower levels (below median) of both glycine and serine, individuals with higher levels (above median) of serine or glycine or both serine and glycine were at reduced risk of pancreatic cancer. The respective odds ratios and 95% Cis were 0.52 (0.31–0.87), 0.38 (0.21–0.70), and 0.24 (0.11–0.63) (Table 4).

We investigated the correlation of serum serine, glycine, and other biomarkers measured with selected demographics, lifestyle factors, and other risk markers among control subjects. Age was positively correlated with serum levels of cysteine and cystathionine whereas BMI, cigarette smoking and alcohol intake were inversely correlated with cysteine and cystathionine (Supplementary Table S2). Smokers had elevated level of glycine (*r* = 0.14, *p* < 0.05). eGFR was inversely correlated with cysteine (*r* = −0.37, *p* < 0.001), cystathionine (*r* = −0.29, *p* < 0.001) and sarcosine (*r* = −0.17, *p* < 0.001). Serum PLP was inversely correlated with serine (*r* = −0.21, *p* < 0.001) and glycine (*r* = −0.26, *p* < 0.001) but positively with cysteine (*r* = 0.26, *p* < 0.001). Among one-carbon biomarkers, the highest correlation was between glycine and serine (*r* = 0.59, *p* < 0.001), followed by cystathionine and sarcosine (*r* = 0.27, *p* < 0.001). All other pairwise correlation coefficients were modest to null (*r’s* < 0.15); Supplementary Table S3).

In the full model, we found that smoking status and education levels were independent risk factors while alcohol drinking per week appeared to be protective factor for pancreatic cancer (Supplementary Table S4). We also performed additional analysis for the association for dietary intakes of protein, energy, fat, and carbohydrates, which were derived from baseline dietary survey at the same time of blood draw, with circulating levels of serine and glycine. No statistically significant association was observed (Table S5).

**Figure 2:**
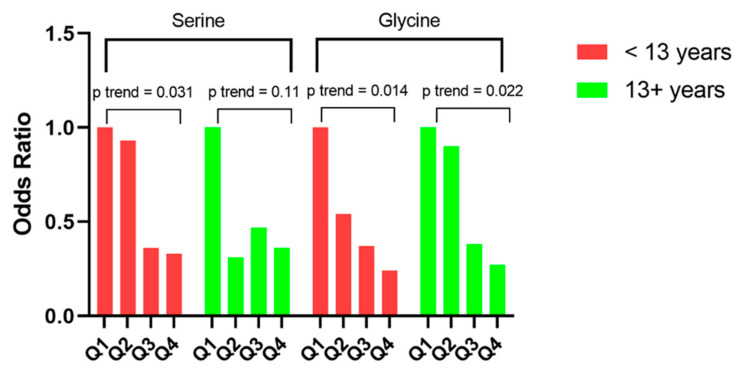
Risk of pancreatic cancer for serum serine and glycine in the Shanghai Cohort Study, stratified by follow-up time.

In stratified analysis by median follow-up time (i.e., <13 versus ≥13 years), the significant inverse trends for serine and glycine with risk of pancreatic cancer were present for both shorter and longer periods of follow-up. Accordingly, the ORs (95% CIs) for the highest relative to the lowest quartiles of serine and glycine were 0.33 (0.09–1.26) and 0.24 (0.07–0.78), respectively, in participants with less than 13 years of follow-up (both *p_trend_* < 0.05). The corresponding figures in participants with 13 or more years of follow-up were 0.36 (0.11–1.14, *p_trend_* = 0.11) and 0.27 (0.07–1.00, *p_trend_* = 0.02), respectively (Figure 2).

## 4. Discussion

In a nested case-control study of 129 pancreatic cancer and 258 free of cancer subjects within the Shanghai Cohort Study of more than 18,000 men living in Shanghai, we found that the lowest risk of pancreatic cancer was observed among individuals with high levels of both serine and glycine. Risk of pancreatic cancer was reduced by more than 70% in individuals with elevated levels of glycine and serine in serum collected, on average, 13 years prior to cancer diagnosis. These strong novel findings, together with our recent results on the inverse association for PLP [36] and methionine [37] with pancreatic cancer risk, suggest that one-carbon metabolism involving serine and glycine may play an important role in the development of pancreatic cancer.

While our result on the inverse association between serine and glycine with risk of pancreatic cancer was inconsistent with a recent study in the Women’s Health Initiative [28], our findings are in line with other studies on lung cancer [23], bladder cancer [24], prostate cancer [25], renal cell carcinoma [26], and colon cancer [29]. The different findings between our study and prior study in the Women’s Health Initiative (WHI) might be due to the difference in the studied populations (Asians vs. Caucasians) or sample size (129 incident pancreatic cancer cases vs. 259 matched controls in our study vs. 30 pairs matched cases-controls in the WHI study [28]).

Methyl donors exert a pivotal effect in genetics, epigenetics and metabolic pathways, and are considered a promising prospect for prevention and risk modification of various diseases including cancer [38]. A number of molecules in methyl-cycles are implicated in tumor formation and progression. The core of this network comprises of interconnected methionine and folate cycles. While nucleotide synthesis is the most relevant end-product of one-carbon cycles and the cornerstone of tumor cells transformation, the other products are playing context-specific roles in tumor transformation [39].

Serine, the major donor of carbon units, is synthesized by phosphoglycerate dehydrogenase (PHGDH) and feeds into the core of one-carbon cycle. Serine is further converted to glycine by serine hydroxymethyltransferase (SHMT) via transferring one carbon to tetrahydrofolate (THF). Recently, Yu et al. [40] reported that an accumulation of upstream glycolytic intermediates may cause increased flux through the serine biosynthesis pathway. Decreased pyruvate production via knocking out the PHGDH gene leads to diminished serine biosynthesis. On the other hand, they [40] revealed serine contributes to pyruvate synthesis in cell lines with knockdown pyruvate kinase M2 (PKM2), an isoform of enzyme highly expressed in pancreatic ductal adenocarcinoma (PDAC) cell [40]. Decreased serine biosynthesis pathway by knocking out the PHGDH gene decreased pyruvate production (i.e., reduced levels of ^13^C-glucose labelled pyruvate), which is highly expressed in PDAC cells, suggesting a protective effect of serine against pancreatic cancer development. Another study by Mohammad et al. [41] in vitro and in vivo preclinical models of pancreatic cancer showed that the treatment with the combination of TEPP-46 (an activator of PKM2) and FX-11 (an inhibitor of lactate dehydrogenase A or LDHA) resulted in increased pyrurate kinase (PK) and reduced LDHA enzyme activity in plasma and tumor tissues, and decreased PKM2 and LDHA expression in tumors and consequently decreased tumor volume and proliferation.

A study from the Vousden’s lab [22], on the other hand, questioned the protective impact of exogenous serine or glycine on tumor growth in autochthonous mouse models of Kras-driven pancreatic cancer. They [22], however, acknowledged that even though the dietary restriction of serine and glycine may be effective in suppressing the development of such cancers, whether the effect of dietary limitation of serine and glycine would change serum or tumor levels of these amino acids in human remains unknown. Further evidence from Amelio et al. indicated the upregulation of the serine/glycine biosynthesis pathway drives oncogenesis [42]. The interaction between the serine/glycine metabolism and tumorigenesis is complicated and a number of genetic and epigenetic factors may interfere with this pathway [43]. The current study might be the first human study to reveal this gap of knowledge by showing that high serum concentrations of serine and glycine many years prior to the clinical diagnosis decreased the risk of pancreatic cancer.

The findings of our study may indicate the specific impact of serine and glycine at the early stages of tumor development. While serine helps tumor progression via activating PMK2 and support metabolic reprogramming of PDAC cells, at the early stages physiologic levels of serine can inhibit tumor development. Hwang et al. [44] reported that serine biosynthesis can induce cancer-protective by-products. A serine biosynthesis by-product such as α-ketoglutarate, a tricarboxylic acid intermediate, functions as a cofactor for dioxygenases involved in regulation of gene expression. Previous study by Morris et al. [45] has shown the effector role of α-ketoglutarate in p-53 tumor suppressive function in pancreatic cancer. Another pathway that might explain the protective impact of glycine on pancreatic cancer may be through the glycine-conjugation of bile acids [46]. Secondary bile acid ursodeoxycholic acid (UCDA), formed by intestinal microbiota, is predominantly conjugated with glycine. Glycine conjugation increases the hydrophilicity with consequent reduced cytotoxicity and membranolytic properties of bile acid [47]. UDCA has antioxidant, anti-inflammatory and cytoprotective properties [48], inhibits cell proliferation by blockade of cell cycle at G1 phase [49]. In addition, Khaire et al. [50] reported that UDCA significantly inhibited *Ras* mutations and Cox-2 protein and mRNA levels in tumors with normal *Ras* activity in colon cancer suggesting its protective effect in the development of colon cancer. Studies in pancreatic cancer has shown the inhibitory effect of UDCA on cancer stem-like cells [51].

Serum concentrations of serine and glycine are reflective of their dietary intake and de novo synthesis. Changes in serine metabolism are influenced by dietary isocaloric protein restriction (i.e., protein malnutrition). Kalhan et al. [52] observed that there was a doubling serine concentration in the blood and livers of the rats fed the protein-restricted diet and 50% increase in the de novo synthesis of this amino acid while there was no change in the concentration of serine in the kidneys of rats fed with protein-restricted diet. Different studies demonstrated that a restricted-protein diet led to increase the concentration of serine in the blood, though the exact mechanism remains to be elucidated [53,54,55]. Furthermore, the consumption of glycine along biosynthesis of glycine have been associated with high-rate proliferating cancer cell lines.

We found that smokers had elevated levels of glycine. Since smoking is a well-known risk factor for pancreatic cancer [8,56,57], we adjusted smoking status in the multivariable regression model so the observed association would be less likely to be confounded by smoking. The association between smoking and glycine might be explained by different pathways. For instance, extensive evidence shows that smokers eat more meat, thus contributing to the evaluated level of glycine. In the COSMOS (Continuous Observation of Smoking Subjects) prospective cohort study of 5203 asymptomatic participants, aged ≥50 years, Gnagnarella et al. [58] found that smoking was positively and statistically significant correlated with red meat (r = 0.048, *p* < 0.05). In a case-control study of 187 lung cancer men and 252 controls in Poland, Hawrysz et al. [59] reported that smokers was positively associated with red meat and processed meat intakes among healthy controls (OR = 2.0, 95% CI: 1.8–2.9 among moderate smokers and OR = 2.0, 95% CI: 1.7–2.2 among heavy smokers). In our own Singapore Chinese Health Study (SCHS) [60], a prospective cohort study of more than 63,000 study participants aged 40–75 years old who are Chinese Singaporeans, we also found that smoking is positively and statistically significant correlated with red meat (r = 0.13, *p* < 0.0001).

The strengths of our study include prospective study design, long-term follow-up, and comprehensive assessment of biomarkers involved in the serine-glycine metabolic pathway using validated state-of-the-art GC-MS/MS and LC-MS/MC assays. In addition, we adjusted for different potential confounders, including eGFR, alcohol consumption, smoking, history of diabetes, PLP, and total methyl donors (i.e., sum of choline, betaine and methionine).

The main limitations of our study are a modest sample size and lack of female subjects, thus limit our ability to generalize our findings to other populations. Given a plausible biological mechanism of one-carbon metabolism in the development of pancreatic cancer, our study with 129 cases and 258 controls would have at least 80% power to detect a minimal odd ratio (OR) of 2 or 0.5 for the two extreme quartiles of biomarker concentrations (https://dceg.cancer.gov/tools/design/power, Accessed date: 15 January 2022). Thus, the present study provided a reasonable effect size of the biomarkers studied on the risk of developing pancreatic cancer. Another limitation is that the examined biomarkers were measured at baseline only. Serum levels of serine and glycine might change over time and these changes would affect to the risk of pancreatic cancer. Further studies are thus warranted to evaluate this association. Furthermore, while we tried to control for potential confounding factors and/or established risk factors for pancreatic cancer in the multivariable model, residual confounding might occur due to the present of unknown factor(s). Finally, our findings are from the nested case-control study within a prospective cohort study of the Chinese Singaporean population, it is thus would not generalizable to other populations.

Our finding on serum serine and glycine in relation to pancreatic cancer risk has several implications and warrants future mechanistic studies. Considering the protective impact of serine and glycine, the crosstalk between genetic profile, nutritional status, and dietary patterns may provide an evidence-based approach for the development of personalized nutrition strategy. The interconnection of one-carbon cycles and the metabolism of glycine and serine prompts a wide variety of targetable enzymes or mediators that can be further investigated for potential chemoprevention of pancreatic cancer.

## 5. Conclusions

In summary, our study found novel and strong, inverse associations between serine and glycine and pancreatic cancer risk in men, supporting a protective role of these two amino acids against the development of pancreatic cancer in humans that might have an implication for cancer prevention.

## Figures and Tables

**Table 1:** Baseline demographic characteristics and lifestyle factors of pancreatic cancer cases and control subjects in the Shanghai Cohort Study.

Baseline Characteristics	Controls	Cases	*p*
Number of subjects	258	129	
Age (years) (Mean ± SD)	56.4 ± 5.5	56.5 ± 5.5	0.74
Body mass index (kg/m^2^) (Mean ± SD)	21.9 ± 2.8	22.5 ± 3.0	0.08
Level of education, *n* (%)			0.36
No formal schooling	13 (5.0)	3 (2.3)	
Primary school	74 (28.7)	34 (26.4)	
Secondary school or above	171 (66.3)	92 (71.3)	
Smoking status, *n* (%)			**0.003**
Never	113 (43.8)	35 (27.1)	
Former	16 (6.2)	6 (4.7)	
Current	129 (50.0)	88 (68.2)	
Cotinine (nmol/L) (Geometric mean ± SD)	440.0 ± 573.8	576.4 ± 573.0	**0.02**
Level of alcohol intake (drinks/week), *n* (%)			0.74
0	146 (56.6)	70 (54.3)	
<7	29 (11.2)	18 (14.0)	
≥7	83 (32.2)	41 (31.8)	
History of diabetes, *n* (%)			0.52
No	254 (98.5)	128 (99.2)	
Yes	4 (1.55)	1 (0.78)	
eGFR (mL/min/1.73 m^2^) (Geometric mean ± SD)	89.7 ± 12.4	91.5 ± 11.3	0.16
PLP (nmol/L) (Geometric mean ± SD)	35.6 ± 46.9	33.9 ± 60.9	0.76
Total methyl donors µmol/L(Geometric mean ± SD) ^a^	114.7 ± 114.2	109.4 ± 112.0	**0.02**

Abbreviations: eGFR, estimated glomerular filtration rate; PLP, pyridoxal 5′-phosphate; SD: standard deviation. ^a^ Total methyl donors: sum of choline, betaine, and methionine. The significant findings are demonstrated in bold fonts.

**Table 2:** Geometric means of serum concentrations of biomarkers studied in pancreatic cancer cases and control subjects in the Shanghai Cohort Study.

Biomarkers * (µmol/L)	Controls, *n* = 258 Geometric Mean (95% CI)	Cases, *n* = 129 Geometric Mean (95% CI)	*p* ^a^
Serine	186.37 (182.98–189.80)	179.01 (174.33–183.80)	**0.017**
Glycine	363.05 (356.96–369.24)	345.52 (337.18–354.00)	**0.002**
Cystathionine	0.28 (0.27–0.30)	0.29 (0.27–0.31)	0.70
Cysteine	272.00 (268.45–275.60)	273.15 (268.01–278.30)	0.73
Sarcosine	1.98 (1.92–2.06)	2.03 (1.93–2.14)	0.49

^a^ *p*-value to compare geometric means adjusted for age and gender, level of education (no formal schooling, primary school, and secondary school or above), body mass index (<18.5, 18.5–<23.0, ≥23.0 kg/m^2^), smoking status (never, former, and current smokers), number of alcoholic drinkers per week (continuous), history of diabetes (no, yes), serum cotinine concentration (nmol/L), serum pyridoxal 5′-phosphate concentration (nmol/L), estimated glomerular filtration rate ((mL/min/1.73 m^2^) and total methyl donors. * Mean serum concentrations. The significant findings are demonstrated in bold fonts.

**Table 3:** Associations between serum concentrations of biomarkers studied and pancreatic cancer risk in the Shanghai Cohort Study.

Biomarkers in Quartile	Controls	Cases	OR (95% CI) ^a^
Serine			
Q1	65	49	1.00
Q2	64	29	0.56 (0.30–1.10)
Q3	65	28	**0.43 (0.22–0.83)**
Q4	64	23	**0.33 (0.14–0.75)**
*p_trend_*			**0.003**
Continuous (log_2_)	258	129	**0.28 (0.09–0.85)**
Glycine			
Q1	65	51	1.00
Q2	64	34	0.68 (0.36–1.27)
Q3	65	23	**0.39 (0.19–0.79)**
Q4	64	21	**0.25 (0.11–0.58)**
*p_trend_*			**0.001**
Continuous (log_2_)	258	129	**0.14 (0.04–0.51)**
Cystathionine			
Q1	65	34	1.00
Q2	64	33	0.94 (0.48–1.83)
Q3	65	30	0.91 (0.46–1.83)
Q4	64	32	1.46 (0.72–2.93)
*p_trend_*			0.77
Continuous (log_2_)	258	129	1.09 (0.75–1.59)
Cysteine			
Q1	65	32	1.00
Q2	64	28	1.05 (0.54–2.06)
Q3	65	38	1.52 (0.78–2.99)
Q4	64	31	1.41 (0.69–2.88)
*p_trend_*			0.26
Continuous (log_2_)	258	129	1.37 (0.31–6.01)
Sarcosine			
Q1	65	34	1.00
Q2	64	29	0.91 (0.47–1.78)
Q3	65	34	1.18 (0.62–2.24)
Q4	64	32	1.27 (0.65–2.47)
*p_trend_*			0.39
Continuous (log_2_)	258	129	1.26 (0.72–2.19)

^a^ Derived from multivariable logistic regression models adjusting for level of education (no formal schooling, primary school, and secondary school or above), body mass index (<18.5, 18.5–<23.0, ≥23.0 kg/m^2^), smoking status (never, former, and current smokers), number of alcoholic drinkers per week (continuous), history of diabetes (no, yes), serum cotinine concentration (nmol/L), serum pyridoxal 5′-phosphate concentration (nmol/L), estimated glomerular filtration rate ((mL/min/1.73 m^2^) and total methyl donors; OR with 95% CI excluding one or *p*_trend_ < 0.05 are in bold.

**Table 4:** Joint effect of serine and glycine on the risk of pancreatic cancer in the Shanghai Cohort Study ^a^.

Glycine	Serine					
	Low (<184.2)		High (≥184.2)		Total	
	Cases/Control	OR (95% CI)	Cases/Control	OR (95% CI)	Cases/Control	OR (95% CI)
Low (<353.3)	61/90	1.00	24/39	0.77 (0.41–1.46)	85/129	1.00
High (≥353.3)	17/39	0.56 (0.26–1.22)	27/90	**0.24 (0.11–0.63)**	44/129	**0.38 (0.21–0.70)**
Total	78/129	1.00	51/129	**0.52 (0.31–0.87)**		

^a^ Derived from multivariable logistic regression models adjusting for level of education (no formal schooling, primary school, and secondary school or above), body mass index (<18.5, 18.5–<23.0, ≥23.0 kg/m^2^), smoking status (never, former, and current smokers), number of alcoholic drinkers per week (continuous), history of diabetes (no, yes), serum cotinine concentration (nmol/L), serum pyridoxal 5′-phosphate concentration (nmol/L), estimated glomerular filtration rate (mL/min/1.73 m^2^) and total methyl donors; ORs with 95% CIs excluding one are in bold. Low and high are defined as individuals with concentrations below and above the median, respectively.

## Data Availability

De-identified data relevant to the report can be shared and is available upon request through the University of Pittsburgh for researchers who meet the criteria for access to confidential data. Data is accessible to the corresponding author and also is available from the University of Pittsburgh Institutional Data Access/Ethics Committee with the following contact information: 3500 Fifth Avenue, Hieber Building Main Office, Suite 106 Pittsburgh, PA 15213. Main Phone: (412) 383-1480. Main Fax: (412) 383-1508. Email: askirb@pitt.edu.

## Supplementary Materials

The following supporting information can be downloaded at: https://www.mdpi.com/article/10.3390/cancers14092199/s1, Table S1: Within-batch and Between-batch Coefficients of Variations (CV) of Biomarkers (*N* = 14); Table S2: title; Spearman Correlation Coefficients Between Serum Biomarkers and Selected Sociodemographic Characteristics among all Control Subjects, Shanghai Cohort Study (*n* = 258); Table S3: Spearman Correlation Coefficients of Serum Biomarkers among all Control Subjects of the Shanghai Cohort Study (*n* = 258); Table S4: Full Models between Serine, Glycine and other Co-variates in relation to Pancreatic Cancer Risk; Table S5: Spearman Correlation Coefficients between Energy, Fat, Protein and Carbohydrate Intakes with Serine and Glycine among Controls, Shanghai Cohort Study (*n* = 258).

## Abbreviations

BMI	body mass index
CI	confidence interval
CV	coefficient of variation
eGFR	estimated glomerular filtration rate
ICD	International Classification of Diseases-Oncology
NF-kB	nuclear factor kappa-light-chain-enhancer of activated B cells or uclear factor kappa B
LC-MS/MS	liquid chromatography-tandem mass spectrometry
OR	odds ratio
PLP	pyridoxal 5′-phosphate
ROS	reactive oxygen species
SCH	Shanghai Cohort Study
SD	standard deviation
