# Supplementary material for: The Association between Serum Serine and Glycine and Related-Metabolites with Pancreatic Cancer in a Prospective Cohort Study
Source: Cancers (Basel). 2022 Apr 28;14(9):2199. doi: 10.3390/cancers14092199 (PMC9105477; doi:10.3390/cancers14092199)
Supplement: Supplementary file 1 [file cancers-14-02199-s001.zip › cancers-1604756-Supplementary.pdf]

cancers MDPI

Supplementary Materials: The Association between Serum Serine and Glycine and Related-Metabolites with Pancreatic Cancer in a Prospective Cohort Study

Hung N. Luu , Pedram Paragomi, Renwei Wang, Joyce Y. Huang, Jennifer Adams-Haduch, Øivind Midttun, Arve Ulvik, Tin C. Nguyen, Randall E. Brand, Yutang Gao, Per Magne Ueland and Jian-Min Yuan

Table S1. Within-batch and Between-batch Coefficients of Variations (CV) of Biomarkers ($N = 14$).

Biomarkers (μmol/L)	Within-batch CV, %	Between-batch CV, %
Serine	0.7	2.0
Glycine	1.0	2.1
Cystathionine	1.4	1.9
Cysteine	5.0	2.6
Sarcosine	0.6	1.3

Table S2. Spearman Correlation Coefficients Between Serum Biomarkers and Selected Sociodemographic Characteristics among all Control Subjects, Shanghai Cohort Study ($n = 258$).

Selected variables	Serine	Glycine	Cystathionine	Cysteine	Sarcosine
Age (years)	−0.10	−0.05	0.15*	0.19*	0.12
Body mass index (kg/m²)	−0.10	−0.18*	0.11	0.22*	−0.04
Smoking status	0.10	0.14*	−0.12*	−0.18*	0.09
Alcohol drinking status	−0.12	0.03	−0.16*	−0.15*	0.15*
Level of education	−0.04	−0.04	0.03	0.13*	−0.03
History of diabetes	0.04	0.11	−0.05	−0.07	−0.07
Serum cotinine (nmol/L)	0.08	0.12	−0.12	−0.16*	0.14*
eGFR (mL/min/1.73m²)	0.09	−0.07	−0.29**	−0.37**	−0.17*
PLP (nmol/L)	−0.21*	−0.26**	0.01	0.26**	−0.03
Total methyl donors	0.40**	0.33**	0.20*	0.01	0.34**

Abbreviations: eGFR, estimated glomerular filtration rate; PLP, pyridoxal 5′-phosphate, $^{*}P < 0.05$, $^{**}P < 0.001$.

Table S3. Spearman Correlation Coefficients of Serum Biomarkers among all Control Subjects of the Shanghai Cohort Study ($n = 258$).

Biomarkers (μmol/L)	Serine	Glycine	Cysteine	Cystathione	Sarcosine
Serine	1.00	**0.59***	−0.04	0.06	0.10
Glycine		1.00	−0.05	0.01	0.12
Cysteine			1.00	−0.02	−0.01
Cystathione				1.00	**0.27***
Sarcosine					1.00

$^{*}P < 0.001$.

Table S4. Full Models between Serine, Glycine and other Co-variates in relation to Pancreatic Cancer Risk.

Variable	OR (95% CI)
Serine	0.78 (0.60–1.03)
Glycine	**0.69 (0.52–0.92)**
Smoking status	**1.73 (1.19–2.52)**
Number of alcoholic drinking per week	0.57 (0.33–0.97)
History of diabetes	2.33 (0.22–24.33)
BMI	1.21 (0.82–1.79)
Education	**1.96 (1.19–3.25)**
Serum cotinine (nmol/L)	1.00 (1.000–1.001)

Serum eGFR (mL/min/1.73 m^2)	1.01 (0.99–1.04)
Serum pyridoxal 5′-phosphate concentration (nmol/L)	0.45 (0.53–1.04)
Total methyl donor (sum of choline, betaine and methinonine)	0.77 (0.56–1.06)

Table S5. Spearman Correlation Coefficients between Energy, Fat, Protein and Carbohydrate Intakes with Serine and Glycine among Controls in the Shanghai Cohort Study (n = 258).

	**Serine**		**Glycine**	
	**Correlation Coefficient**	***P*-value**	**Correlation Coefficient**	***P*-value**
Energy intake (Kcal/day)	−0.03248	0.60	0.06031	0.33
Fat intake (g/day)	−0.02358	0.71	0.01287	0.84
Protein intake (g/day)	−0.02180	0.73	−0.05174	0.41
Carbohydrate intake (g/day)	−0.06022	0.33	−0.01978	0.75
